# The value of myocardial work in assessment of ventricular function in patients with non-obstructive hypertrophic cardiomyopathy

**DOI:** 10.1186/s12872-022-02740-4

**Published:** 2022-07-05

**Authors:** Chenguang Yang, Ying Guo, Xiang Wang, Ruisheng Zhang, Fang Wang, Huolan Zhu

**Affiliations:** 1 Department of Cardiology, Beijing Hospital, National Center of Gerontology, Institute of Geriatric Medicine, Chinese Academy of Medical Sciences, No.1 Dahua Road, Dongdan, Dongcheng District, Beijing, 100730 China; 2 Department of Gerontology, Shaanxi Provincial People’s Hospital, Shaanxi Provincial Clinical Research Center for Geriatric Medicine, No.256 Youyi West Road, Xi’an, China

**Keywords:** Echocardiography, Hypertrophic cardiomyopathy, Myocardial work, Speckle tracking

## Abstract

**Background:**

To evaluate myocardial work using speckle tracking echocardiography in patients with non-obstructive hypertrophic cardiomyopathy (HCM).

**Methods:**

Fifty patients with HCM and 50 normal controls were included. Left ventricular ejection fraction (LVEF) was quantified using the bi-plane Simpson’s method. Myocardial work parameters, which included global work index (GWI), global constructive work (GCW), global waste work (GWW), and global work efficiency (GWE), were derived from the 2D strain-pressure loop.

**Results:**

The patient group was older (49.19 ± 14.69 vs. 37.16 ± 7.49 years old) and had a higher body mass index (24.93 ± 3.67 vs. 23.26 ± 3.32 kg/m^2^) and systolic blood pressure (121.81 ± 16.50 vs. 115.30 ± 11.01 mmHg) (*P* < 0.05). The mean LVEF in patients was 51%, with 54% of patients had LVEF ≤ 50%. Compared to controls, GWI (946.42 ± 360.64 vs. 1639.72 ± 204.56 mmHg%), GCW (1176.94 ± 373.23 vs. 1960.16 ± 255.72 mmHg%), and GWE (83.96 ± 7.68 vs. 95.26 ± 1.98%) were significantly decreased, while GWW (158.17 ± 82.47 vs. 79.12 ± 40.26 mmHg%) was significantly increased (*P* < 0.05) in the patient group. In patients, GWE showed a trend of positive correlation with LVEF (r = 0.276, *P* = 0.06), while GWW had a trend of negative correlation with LVEF (r = − 0.241, *P* = 0.09). No correlation between myocardial work and LV diastolic function or QRS duration was observed. Maximal wall thickness significantly correlated with all the myocardial work parameters.

**Conclusions:**

Assessing myocardial work adds useful information of LV function in patients with non-obstructive HCM.

## Background

Echocardiography plays an important role in the diagnosis and treatment of patients with hypertrophic cardiomyopathy (HCM) [1]. The conventional echocardiographic parameters for HCM assessment include left ventricular wall thickness, left ventricular systolic and diastolic function, Doppler gradient across the left ventricular outflow tract, the motion of mitral valve, size/volume of left atrium and right ventricle, etc. [2]. However, conventional parameters, such as left ventricular ejection fraction (LVEF), may also be impacted by factors other than the myocardial damage [3]. Evidence shows that such damage exists and is often underestimated in HCM patients with a normal LVEF [4]. Once the disease progresses and the LVEF drops below 50%, myocardial damage in these patients may quickly worsen [5].

Previous studies revealed that the circumferential contraction of the left ventricle in patients with HCM is preserved at an early stage. This can explain a normal or increased LVEF or left ventricular shortening fraction in HCM patients [6]. However, the longitudinal function of the left ventricle, e.g. global longitudinal strain (GLS), in HCM patients is significantly reduced when assessed by echocardiography speckle tracking [6]. Similarly, at an early stage of HCM, the systolic and diastolic tissue Doppler imaging (TDI) velocities can be reduced when other functional parameters are still in the normal range [7]. TDI velocities have a good diagnostic value for identifying mutation-positives without left ventricular hypertrophy [7].

Although considered better than other echocardiographic parameters, both GLS and TDI velocities are inevitably impacted by ventricular afterload and therefore have their limitations [8]. In patients with elevated ventricular afterload but no significant myocardial damage, these indices may be reduced therefore leading to a false positive finding. Unlike GLS, myocardial work has included the afterload information through generating a pressure-strain loop (PSL). Myocardial work can be used to assess the myocardial damage relatively independent of ventricular afterload [4, 9]. Studies have found the reduction in global constructive work (GCW) in patients with HCM was associated with left ventricular fibrosis [10].

Currently, there are limited studies about myocardial work in patients with HCM. The aim of this study was to investigate myocardial work in patients with HCM using speckle tracking echocardiography and assess the association between LVEF and myocardial work.

## Methods

### Subjects

Fifty patients with HCM and 50 normal controls were included in the study. Normal controls were recruited from subjects who came to the hospital for a physical examination. The control subjects did not have any symptoms of cardiovascular diseases or any other diseases, nor did they have history of chronic diseases or drug treatment. Patients were consecutively recruited in the study. HCM was diagnosed if the wall thickness of any left ventricular segment was ≥ 15 mm as determined by echocardiography or magnetic resonance imaging, and left ventricular hypertrophy was not secondary to any specific etiology [5]. Patients were excluded if the peak Doppler gradient across the left ventricular outflow tract was ≥ 30 mmHg or intra-ventricular Doppler gradient/obstruction was observed. Patients were also excluded if they had coronary heart disease, malignant arrythmia, moderate to severe valvular disease, congenital heart disease, or poor image quality from echocardiography. All participants signed informed consent forms. The study complied with the Declaration of Helsinki and was approved by the ethics committee of Beijing Hospital.

### Conventional echocardiography

General characteristics including age, gender, height and weight, habit of smoking, and baseline diseases (coronary heart disease, hypertension, diabetes, etc.), were collected. All participants received an echocardiographic examination using GE vivid E9 (GE, Horten, Norway) with a 3.5 Hz probe M5S. Patients were lying quietly on their left sides and were connected to an ECG during the scan. The wall thickness of interventricular septum (IVSd) and left ventricular posterior wall (LVPWd) and the left ventricular enddiastolic diameter (LVDd) were measured from the parasternal long axis view of the left ventricle at its end-diastole. The peak velocities of mitral valve early diastolic (E) and late diastolic (A) waves were measured using Doppler with a sample volume positioned 1 cm below the mitral valve annulus (at the tip of the mitral valve). The E/A ratio was calculated. Left ventricular E/E’ was the ratio between mitral valve Doppler E velocity and TDI E’ velocity at the mitral valve annulus. Based on the guidelines from the American Society of Echocardiography [11], LVEF was derived using the bi-plane Simpson’s method.

### Speckle tracking

Myocardial work parameters were measured using the speckle tracking technique. Apical four, two and three chamber views were obtained with frame rates between 45 and 75 from all participants. The offline analysis was done on EchoPAC V203 (GE, Chicago, IL). After defining the sample volume along the myocardial wall, visual inspection was used to ensure sufficient tracking. Myocardial work parameters were obtained by generating a PSL. The x-axis was for strain and y-axis for ventricular afterload. One loop corresponded to one cardiac cycle. Based on published studies [12], brachial artery blood pressure was used to replace the left ventricular pressure. Global work index (GWI) was the area on PSL between mitral valve closure and mitral valve opening. GCW was the myocardial work used for its shortening during ventricular systole and for lengthening during isovolumic relaxation. Global waste work (GWW) was the myocardial work wasted for lengthening during ventricular systole and for shortening during isovolumic relaxation. Global work efficiency (GWE) was the ratio between GCW and the total work (sum of GCW and GWW).

### Statistics

SPSS 25 (IBM Corp., Armonk, NY, USA) was used for analysis. Visual inspection and Shapiro–Wilk test were applied to confirm the normality of the data distribution. Categorical data are presented as percentage (%) and continuous data are described as mean ± standard deviation. Depending on the type of data, the Chi-square or ANOVA test was used to compare the difference between groups. Pearson or Spearman correlation was used based on the distribution of continuous data. A *P*-value < 0.05 was regarded as significant.

## Results

### General characteristics (Table 1)

**Table 1:** General characteristics of patients with HCM and normal controls

	Normal controls (n = 50)	Patients (n = 50)
Age (yrs)	37.16 ± 7.49	49.19 ± 14.69*
BSA (m^2^)	1.85 ± 0.19	1.87 ± 0.19
BMI (kg/m^2^)	23.26 ± 3.32	24.93 ± 3.67*
SBP (mmHg)	115.30 ± 11.01	121.81 ± 16.50*
DBP (mmHg)	73.78 ± 1.37	74.94 ± 10.60
Male, case (%)	30 (60)	26 (52)
QRS duration, (ms)	86.14 ± 6.58	98.80 ± 17.85*

*BSA* body surface area; *BMI* body mass index; *DBP* diastolic blood pressure; *SBP* systolic blood pressure. *P < 0.05

Patients with HCM were significantly older than the healthy controls (49 ± 15 vs. 37 ± 7 years, *P* < 0.05). Patients had significantly higher body mass index (BMI) (25 ± 4 vs. 23 ± 3 kg/m^2^, *P* < 0.05), but there was no difference in body surface area (BSA) between the groups. There was significantly higher systolic blood pressure in patients than normal controls (122 ± 17 vs. 115 ± 11 mmHg, *P* < 0.05). HCM patients had an QRS duration on ECG 98.80 ± 17.85 ms while in controls the QRS duration was 86.14 ± 6.58 ms (*p* < 0.05). In patients there were complete right bundle branch block (CRBBB) in 2, CRBBB with left anterior bundle branch block in 2, complete left bundle branch block in 1, intraventricular block in 3, first degree atrioventricular block in 2. No patients received pacemaker.

### Conventional echocardiography (Table 2)

**Table 2:** Conventional echocardiography for patients with HCM and normal controls

	Normal controls (n = 50)	Patients (n = 50)
IVSd (mm)	9.18 ± 1.51	15.87 ± 5.88*
PWd (mm)	8.85 ± 1.43	9.93 ± 3.23*
LVDd (mm)	43.54 ± 4.23	43.29 ± 4.28
LVEDV (ml)	86.62 ± 19.47	84.88 ± 26.96*
LVEF (%)	0.64 ± 0.03	0.51 ± 0.06*
Doppler A wave at mitral valve (cm/s)	0.62 ± 0.14	0.70 ± 0.28*
Doppler E wave at mitral valve (m/s)	0.81 ± 0.13	0.64 ± 0.17
E/A ratio	1.39 ± 0.46	1.05 ± 0.49
Tissue Doppler E’ wave at MV annulus (m/s)	0.13 ± 0.03	0.04 ± 0.01*
E/E’ ratio	6.59 ± 1.37	16.59 ± 6.64*

*IVSd* thickness of interventricular septum at end-diastole; *LVDd* left ventricular dimension at end-diastole; *LVEDV* left ventricular volume at end-diastole; *LVEF* left ventricular ejection fraction; *PWd* thickness of posterior wall at end-diastole. *P < 0.05

There were three patients had apical hypertrophy. The IVSd and LVPWd were significantly higher in patients than in normal controls (*P* < 0.05). There was no significant difference in LVDd between the two groups. Compared to normal controls, the left ventricular end-diastolic volume (LVEDV) and the LVEF were significantly lower in the patients (*P* < 0.05). Left ventricular TDI E’ was significantly lower and the E/E’ ratio was significantly higher in patients than in controls (*P* < 0.05). There was no significant difference in E/A ratio between the groups.

### Speckle tracking

**Fig. 1:**
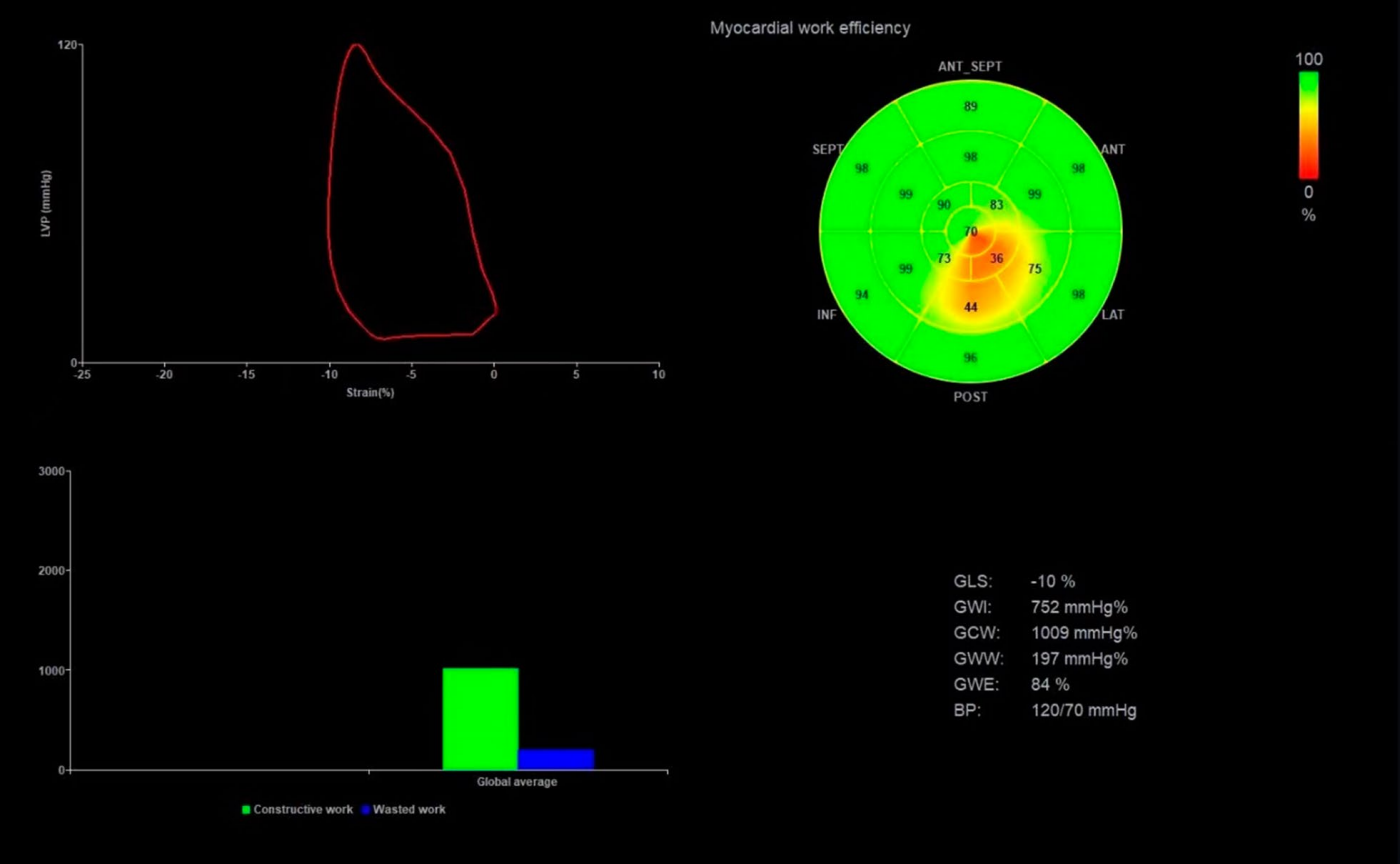
Myocardial work in a patient with HCM. Top left: pressure-strain loop; Top right: bull’s eye of LV segmental myocardial work; Bottom left: comparison diagram of constructive work and waste work. Bottom right: parameters regarding myocardial work. LV: left ventricle; HCM: hypertrophic cardiomyopathy

**Fig. 2:**
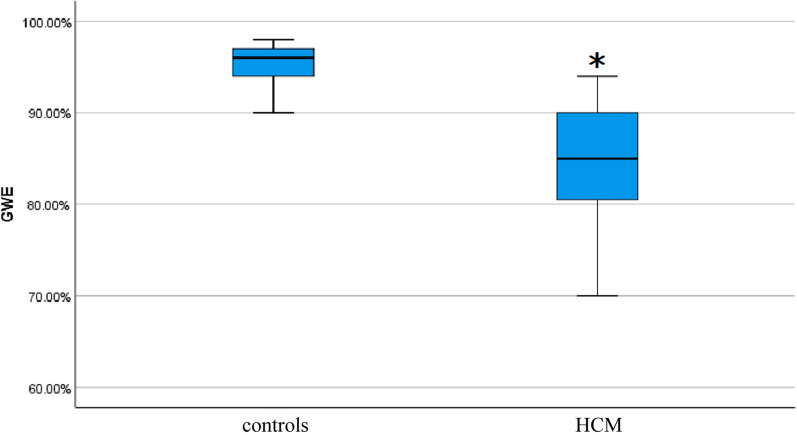
Comparing GWE between patients with HCM and normal controls. Boxplots showing that the median value of GWE was significantly lower in patients with HCM. GWE: global work efficiency; HCM: hypertrophic cardiomyopathy. *P < 0.05

**Fig. 3:**
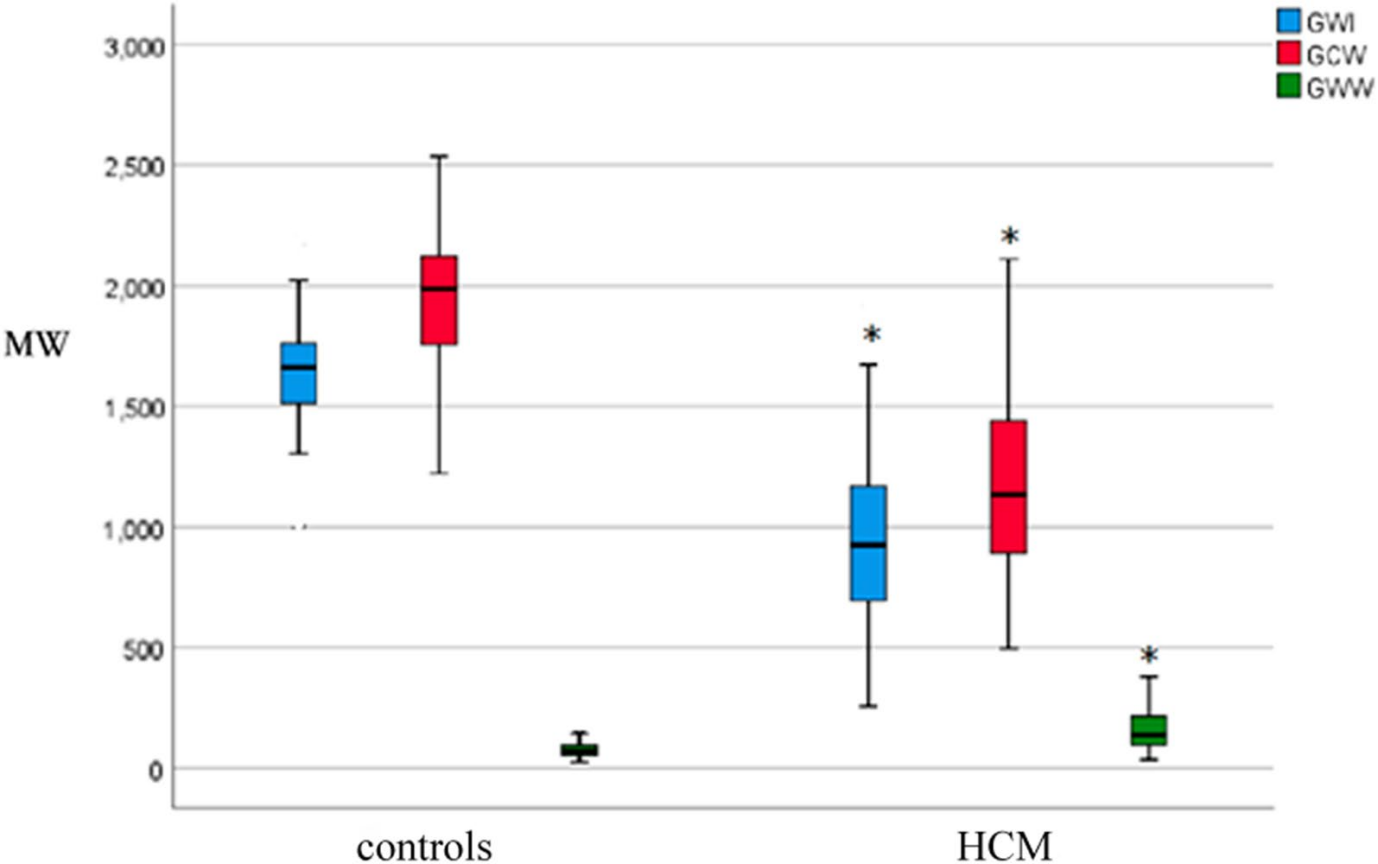
Comparing myocardial work parameters between patients with HCM and normal controls. Boxplots showing that the median values of GCW and GWI were significantly lower and the median value of GWW was significantly higher in patients with HCM. GCW: Global constructive work; GWI: Global work index; GWW: Global wasted work; HCM: hypertrophic cardiomyopathy. *P < 0.05

In patients with HCM, the PSL was shifted to the right (Fig. 1). Compared to normal controls, GWI (946.42 ± 360.64 vs. 1639.72 ± 204.56 mmHg%, *P* < 0.05), GCW (1176.94 ± 373.23 vs. 1960.16 ± 255.72 mmHg%, *P* < 0.05), and GWE (83.96 ± 7.68 vs. 95.26 ± 1.98%, *P* < 0.05) were significantly decreased, while GWW (158.17 ± 82.47 vs. 79.12 ± 40.26 mmHg%, *P* < 0.05) significantly increased in the patients (Table 3, Figs. 2 and 3). Overall, myocardial work parameters had a weak or no correlation with LVEF (Table 4). GWE showed a significant positive correlation with LVEF, while GWW had a significant negative correlation with LVEF.

**Table 3:** Parameters of myocardial work for patients with HCM and normal controls

	Normal controls (n = 50)	Patients with HCM (n = 50)
GWE (%)	95.26 ± 1.98	83.96 ± 7.68*
GWI (mmHg%)	1639.72 ± 204.56	946.42 ± 360.64*
GCW (mmHg%)	1960.16 ± 255.72	1176.94 ± 373.23*
GWW (mmHg%)	79.12 ± 40.26	158.17 ± 82.47*

*GCW* Global constructive work; *GWE* Global work efficiency; *GWI* Global work index; *GWW* Global wasted work, *HCM* hypertrophic cardiomyopathy. *P < 0.05

**Table 4:** Correlations between myocardial work parameters and LVEF

	Coefficient	p-value
GWE	0.276	0.06
GWI	0.175	0.23
GCW	0.143	0.32
GWW	− 0.241	0.09

*GCW* Global constructive work; *GWE* Global work efficiency; *GWI* Global work index; *GWW* Global wasted work; *LVEF* left ventricular ejection fraction

## Correlations between myocardial work parameters and wall thickness/LV functions/QRS duration

Maximal wall thickness had significant correlations with GWE (r = -− 0.558, *p* < 0.001), GWI (r = -− 0.387, *p* = 0.007), GCW (r = -− 0.359, *p* = 0.012) and GWW (r = 0.36, *p* = 0.012) (Table 5). No significant correlations were observed between LV diastolic performance or QRS duration and any of the myocardial work parameters.

**Table 5:** Correlations between myocardial work parameters and LV wall thickness

	Coefficient	p-value
GWE	− 0.558	0.000
GWI	− 0.387	0.007
GCW	− 0.359	0.012
GWW	0.36	0.012

*GCW* Global constructive work; *GWE* Global work efficiency; *GWI* Global work index; *GWW* Global wasted work

## Discussion

Our study found that (1) compared to normal controls, patients with HCM had significantly decreased GCW, GWE, and GWI, and increased GWW, and (2) in patients with HCM, the myocardial work-related parameters had weak or no correlation with LVEF. This is the first report from a Chinese population. Similar findings have been observed in other cohorts [10, 13].

Although ventricular stroke volume in patients with HCM is not increased, the myocardial work used for ventricular pumping is increased due to left ventricular outflow tract obstruction [14]. However, even in patients who do not have left ventricular outflow tract obstruction myocardial work efficiency is significantly lower than in controls [13]. This indicates that mechanisms other than afterload increase are involved. Abnormal myocardial fiber alignment and interstitial fibrosis exist in patients with HCM, which impact left ventricular systolic and diastolic function [15]. Because the impact of myocardial fibrosis and abnormal fiber alignment on left ventricular function starts at a pre-clinical stage, conventional echocardiography may not be able to diagnose the pathological process when it first starts. Once patients present with LVEF less than 50%, their situation may deteriorate quickly [2]. Thus, quantifying the longitudinal change using speckle tracking becomes important as it can help identify pre-clinical myocardial damage in the left ventricle [16]. Our study found that both GWE and GWW significantly changed in HCM patients with normal LVEF. None of the myocardial work-related parameters showed a good correlation with LVEF. Hiemstra et al. found only a weak correlation between GCW and left ventricular diastolic function [13]. Thus, the findings presented here, as well as in previous studies, support that the myocardial work-related parameters may be better tools for early diagnosis in HCM patients.

In patients with apical hypertrophy, the apical segment suffers the greatest reduction in GCW, while the apical GCW is preserved in septal type HCM [13]. Abnormal myocardial work appears to be highly specific to the myocardial region where hypertrophic cardiomyopathy is involved. A recent study showed regional myocardial work has great potential for non-invasively predicting high-risk patients with stable coronary heart disease and normal wall motion and preserved left ventricular function [17]. Myocardial work is derived from 2D strain, and although myocardial strain has been applied widely [18], the observer variability of segmental strain is a well-known disadvantage compared with the global strain [19]. We believe myocardial work derived from strain is inevitably impacted by segmental variability. Therefore, in the current study we only reported global myocardial work parameters.

There are some potential impacts on the difference of myocardial work parameters observed between the two groups. In our study, the mean age of the patient group was higher than the control group. A previous study showed that normal people had an upward shift to further stable values of GCW and a linear increase of GWW with advancing age, resulting in lower GWE [20]. However, such an age-related difference will be much milder in HCM patients, as Morbach et al. found that the GCW change per 10 years was 25–51 mmHg% depending on if the subject was ≤ 45 years old. The difference of the mean GCW we observed between HCM patients and controls was close to 800 mmHg%. This certainly cannot be explained by a difference of mean age of ~ 12 years. We also notice a mild increase in BMI in our patient group. The impact of obesity on the heart is complex and involves multiple pathways. Obese patients may have high cholesterol levels, high blood pressure, and/or diabetes, all of which can compromise myocardial performance [21]. With a BMI of 24.93 ± 3.67 kg/m^2^, a majority of our patients did not meet the criteria for obesity. Thus, we propose that a mild increase in BMI does not play a significant role in deciding myocardial work. Finally, our patients had slightly higher systolic blood pressure. It is known that high blood pressure can lead to left ventricular hypertrophy. In stage 1 and stage 2 hypertensive patients, myocardial work is elevated [22]. This helps to differentiate between HCM and hypertension, although both have left ventricular hypertrophy.

Speckle tracking derived myocardial work not only can reveal myocardial damage in HCM patients [23], but can also be used to predict prognosis in this group [24, 25]. Patients with HCM have a relatively benign prognosis [26]. Still, patients can experience death, arrythmia, and other common symptoms [27]. Left ventricular wall thickness is one of the significant predictors for cardiovascular death in HCM patients [27]. Since a thick myocardium is linked with abnormal myocardial work, the prognostic value of myocardial work parameters in predicting the prognosis deserves further investigation.

The current included fifty patients. This may be why we didn’t observe the wide correlations between myocardial constructive work and LV diastolic function in 110 patients. However maximum LV wall thickness did have negative correlations with GWE/GWI/GCW and a positive correlation with GWW. This may indicate maximal wall thickness is a stronger predictor for reduced myocardial work in HCM patients. Maximal wall thickness is known for its prognostic value [28]. The correlations between maximal wall thickness with myocardial work further support it role in clinical echocardiography.

HCM patients suffer mechanical dyssynchrony [29] which is associated with myocardial work [30]. However, we didn’t observe the correlation between QRS duration and myocardial work. Whether QRS duration is a good surrogate for LV mechanical desynchrony is controversial [31]. In our cohort, only three patients had confirmed apical hypertrophy. Study shows mechanical dyssynchrony are often seen in HCM patients on apical form [32]. This can be another explanation we didn’t observed the correlation.

### Limitations

Our findings are derived from a single center with small sample size. Large scale and multi-center studies are needed to further investigate the clinical value of myocardial work. Normal reference data for the Chinese population are missing. In patients with poor image quality, myocardial work is not a suitable method. Just like strain analysis [33], inter-vendor variability may exist for myocardial work. Gender may significantly contribute to variation of normal values of myocardial work [34]. However, in the current study we did not match gender between the two groups. Same for the age difference between the patients and controls.

## Conclusions

Measuring myocardial work is feasible and adds useful information of left ventricular function. Myocardial work has the potential to become a new risk stratifying tool in non-obstructive HCM patients.

## Data Availability

The datasets used and/or analyzed during the current study are available from the corresponding author on reasonable request.

## Abbreviations

A: Late diastolic
BMI: Body mass index
BSA: Body surface area
E: Early diastolic
GCW: Global constructive work
GLS: Global longitudinal strain
GWE: Global work efficiency
GWI: Global work index
GWW: Global waste work
HCM: Hypertrophic cardiomyopathy
IVSd: Interventricular septum
LVDd: Left ventricular enddiastolic diameter
LVEDV: Left ventricular end-diastolic volume
LVEF: Left ventricular ejection fraction
LVPWd: Left ventricular posterior wall
PSL: Pressure-strain loop
TDI: Tissue Doppler imaging

## References

[1] Mandeş L, Roşca M, Ciupercă D, Popescu BA (2020). The role of echocardiography for diagnosis and prognostic stratification in hypertrophic cardiomyopathy. J Echocardiogr.

[2] Dominguez F, González-López E, Padron-Barthe L, Cavero MA, Garcia-Pavia P (2018). Role of echocardiography in the diagnosis and management of hypertrophic cardiomyopathy. Heart.

[3] Marwick TH (2018). Ejection fraction pros and cons: JACC state-of-the-art review. J Am Coll Cardiol.

[4] Vigneault DM, Yang E, Jensen PJ, Tee MW, Farhad H, Chu L (2019). Left ventricular strain is abnormal in preclinical and overt hypertrophic cardiomyopathy: cardiac MR feature tracking. Radiology.

[5] Elliott PM, Anastasakis A, Borger MA, Borggrefe M, Cecchi F, Charron P (2014). 2014 ESC guidelines on diagnosis and management of hypertrophic cardiomyopathy: the task force for the diagnosis and management of hypertrophic cardiomyopathy of the European society of cardiology (ESC). Eur Heart J.

[6] Serri K, Reant P, Lafitte M, Berhouet M, Le Bouffos V, Roudaut R (2006). Global and regional myocardial function quantification by two-dimensional strain: application in hypertrophic cardiomyopathy. J Am Coll Cardiol.

[7] Nagueh SF, Bachinski LL, Meyer D, Hill R, Zoghbi WA, Tam JW (2001). Tissue Doppler imaging consistently detects myocardial abnormalities in patients with hypertrophic cardiomyopathy and provides a novel means for an early diagnosis before and independently of hypertrophy. Circulation.

[8] Rhea IB, Rehman S, Jarori U, Choudhry MW, Feigenbaum H, Sawada SG (2016). Prognostic utility of blood pressure-adjusted global and basal systolic longitudinal strain. Echo Res Pract.

[9] Russell K, Eriksen M, Aaberge L, Wilhelmsen N, Skulstad H, Gjesdal O (2013). Assessment of wasted myocardial work: a novel method to quantify energy loss due to uncoordinated left ventricular contractions. Am J Physiol Heart Circ Physiol.

[10] Galli E, Vitel E, Schnell F, Le Rolle V, Hubert A, Lederlin M (2019). Myocardial constructive work is impaired in hypertrophic cardiomyopathy and predicts left ventricular fibrosis. Echocardiography.

[11] Lang RM, Badano LP, Mor-Avi V, Afilalo J, Armstrong A, Ernande L (2015). Recommendations for cardiac chamber quantification by echocardiography in adults: an update from the American society of echocardiography and the European association of cardiovascular imaging. J Am Soc Echocardiogr.

[12] Russell K, Eriksen M, Aaberge L, Wilhelmsen N, Skulstad H, Remme EW, Haugaa KH, Opdahl A, Fjeld JG, Gjesdal O (2012). A novel clinical method for quantification of regional left ventricular pressure–strain loop area: A non-invasive index of myocardial work. Eur Heart J..

[13] Hiemstra YL, van der Bijl P, El Mahdiui M, Bax JJ, Delgado V, Marsan NA (2020). Myocardial work in nonobstructive hypertrophic cardiomyopathy: implications for outcome. J Am Soc Echocardiogr.

[14] Timmer SA, Germans T, Götte MJ, Rüssel IK, Dijkmans PA, Lubberink M (2010). Determinants of myocardial energetics and efficiency in symptomatic hypertrophic cardiomyopathy. Eur J Nucl Med Mol Imaging.

[15] Ho CY, López B, Coelho-Filho OR, Lakdawala NK, Cirino AL, Jarolim P (2010). Myocardial fibrosis as an early manifestation of hypertrophic cardiomyopathy. N Engl J Med.

[16] Wang A, Cabreriza SE, Cheng B, Shanewise JS, Spotnitz HM (2014). Feasibility of speckle-tracking echocardiography for assessment of left ventricular dysfunction after cardiopulmonary bypass. J Cardiothorac Vasc Anesth.

[17] Zhang J, Liu Y, Deng Y, Zhu Y, Sun R, Lu S (2021). Non-invasive global and regional myocardial work predicts high-risk stable coronary artery disease patients with normal segmental wall motion and left ventricular function. Front Cardiovasc Med.

[18] Smiseth OA, Torp H, Opdahl A, Haugaa KH, Urheim S (2016). Myocardial strain imaging: how useful is it in clinical decision making?. Eur Heart J.

[19] Mirea O, Pagourelias ED, Duchenne J, Bogaert J, Thomas JD, Badano LP (2018). Variability and reproducibility of segmental longitudinal strain measurement: a report from the EACVI-ASE strain standardization task force. JACC Cardiovasc Imaging.

[20] Morbach C, Sahiti F, Tiffe T, Cejka V, Eichner FA, Gelbrich G (2020). Myocardial work - correlation patterns and reference values from the population-based STAAB cohort study. PLoS ONE.

[21] Powell-Wiley TM, Poirier P, Burke LE, Després JP, Gordon-Larsen P, Lavie CJ (2021). Obesity and cardiovascular disease: a scientific statement from the American heart association. Circulation.

[22] Jaglan A, Roemer S, Perez Moreno AC, Khandheria BK (2021). Myocardial work in Stage 1 and 2 hypertensive patients. Eur Heart J Cardiovasc Imaging.

[23] Almaas VM, Haugaa KH, Strøm EH, Scott H, Smith HJ, Dahl CP (2014). Noninvasive assessment of myocardial fibrosis in patients with obstructive hypertrophic cardiomyopathy. Heart.

[24] Hiemstra YL, Debonnaire P, Bootsma M, van Zwet EW, Delgado V, Schalij MJ (2017). Global longitudinal strain and left atrial volume index provide incremental prognostic value in patients with hypertrophic cardiomyopathy. Circ Cardiovasc Imaging.

[25] Haland TF, Almaas VM, Hasselberg NE, Saberniak J, Leren IS, Hopp E (2016). Strain echocardiography is related to fibrosis and ventricular arrhythmias in hypertrophic cardiomyopathy. Eur Heart J Cardiovasc Imaging.

[26] Ten Cate FJ (1996). Prognosis of hypertrophic cardiomyopathy. J Insur Med.

[27] Liu Q, Li D, Berger AE, Johns RA, Gao L (2017). Survival and prognostic factors in hypertrophic cardiomyopathy: a meta-analysis. Sci Rep.

[28] Bois JP, Geske JB, Foley TA, Ommen SR, Pellikka PA (2017). Comparison of maximal wall thickness in hypertrophic cardiomyopathy differs between magnetic resonance imaging and transthoracic echocardiography. Am J Cardiol.

[29] Tsai SY, Wang SY, Shiau YC, Wu YW (2018). Mechanical dyssynchrony and diastolic dysfunction are common in LVH: a pilot correlation study using Doppler echocardiography and CZT gated-SPECT MPI. Sci Rep.

[30] Liu W, Hu C, Wang Y, Cheng Y, Zhao Y, Liu Y (2021). Mechanical synchrony and myocardial work in heart failure patients with left bundle branch area pacing and comparison With biventricular pacing. Front Cardiovasc Med.

[31] Andrikopoulos GK, Tzeis S, Kolb C, Sakellariou D, Avramides D, Alexopoulos EC (2009). Correlation of mechanical dyssynchrony with QRS duration measured by signal-averaged electrocardiography. Ann Noninvasive Electrocardiol.

[32] Tsai SY, Wu YW (2017). Mechanical dyssynchrony and diastolic dysfunction are common in hypertrophic cardiomyopathy: a correlation study using Doppler echocardiography and CZT gated SPECT. J Nucl Med.

[33] Nagata Y, Takeuchi M, Mizukoshi K, Wu VC, Lin FC, Negishi K (2015). Intervendor variability of two-dimensional strain using vendor-specific and vendor-independent software. J Am Soc Echocardiogr.

[34] Truong VT, Vo HQ, Ngo TNM, Mazur J, Nguyen TTH, Pham TTM (2022). Normal ranges of global left ventricular myocardial work indices in adults: a meta-analysis. J Am Soc Echocardiogr.

